# Diagnostic value of cerebrospinal fluid Neutrophil Gelatinase-Associated Lipocalin for differentiation of bacterial meningitis from tuberculous meningitis or cryptococcal meningitis: a prospective cohort study

**DOI:** 10.1186/s12967-023-04485-w

**Published:** 2023-09-07

**Authors:** Qi Wang, Qingwen Lin, Haiyan Wang, Minjie Tang, Kengna Fan, Zeqin Zhang, Er Huang, Weiqing Zhang, Fengqing Wang, Qishui Ou, Xiaofeng Liu

**Affiliations:** 1 Department of Laboratory Medicine, Gene Diagnosis Research Center, The First Affiliated Hospital, Fujian Medical University, Fuzhou, 350005 China; 2 Department of Laboratory Medicine, National Regional Medical Center, Binhai Campus of the First Affiliated Hospital, Fujian Medical University, Fuzhou, 350212 China; 3 Fujian Key Laboratory of Laboratory Medicine, The First Affiliated Hospital, Fujian Medical University, Fuzhou, 350005 China; 4 Fujian Clinical Research Center for Clinical Immunology Laboratory Test, the First Affiliated Hospital, Fujian Medical University, Fuzhou, 350005 China

**Keywords:** Bacterial meningitis, Neutrophil Gelatinase-Associated Lipocalin, Cerebrospinal fluid, Diagnostic biomarker, Central nervous system infection

## Abstract

**Background:**

The early differential diagnosis between bacterial meningitis (BM) and tuberculous meningitis (TBM) or cryptococcal meningitis (CM) remains a significant clinical challenge. Neutrophil Gelatinase-Associated Lipocalin (NGAL) has been reported as a novel inflammatory biomarker in the early stages of infection. This study aimed to investigate whether cerebrospinal fluid (CSF) NGAL can serve as a potential biomarker for distinguishing between BM and TBM or CM.

**Methods:**

We prospectively enrolled the patients with suspected CNS infections at admission and divided them into three case groups: BM (n = 67), TBM (n = 55), CM (n = 51), and an age- and sex-matched hospitalized control (HC, n = 58). Detected the CSF NGAL and assessed its diagnostic accuracy in distinguishing between BM and TBM or CM. Additionally, longitudinally measured the CSF NGAL levels in patients with BM to evaluate its potential as a monitoring tool for antibacterial treatment.

**Results:**

The concentration of CSF NGAL in BM was significantly higher than in TBM, CM, and HC (all* P* < 0.05), while the serum NGAL did not show significant differences among the three case groups. The ROC analysis demonstrated that CSF NGAL presented a good diagnostic performance with an AUC of 0.834 (0.770–0.886) and at the optimal cutoff value of 74.27 ng/mL with 70.15% sensitivity and 77.36% specificity for discriminating BM with TBM and CM. Additionally, the CSF NGAL in the convalescent period of BM was significantly lower than in the acute period (*P* < 0.05).

**Conclusions:**

CSF NGAL may serve as a potential biomarker for distinguishing between acute BM and TBM or CM. Additionally, it holds clinical significance in monitoring the effectiveness of antibiotic therapy for BM.

**Supplementary Information:**

The online version contains supplementary material available at 10.1186/s12967-023-04485-w.

## Introduction

Central nervous system (CNS) infection, commonly presenting as meningitis or encephalitis, is a serious medical condition with high mortality and morbidity. Even survivors may experience significant disability due to neurological deficits [1, 2]. As delayed initiation of antibiotic treatment is closely related to poor outcomes (high disability and mortality), prompt recognition and treatment of CNS infections are crucial [3–5]. However, the early diagnosis of many CNS infections is challenging due to non-specific clinical manifestations and insensitive laboratory tests. As a result, empiric therapy is often initiated without identifying the causative pathogen, leading to inappropriate treatment.

Conventional diagnostic technologies for CNS infections, such as the cerebrospinal fluid (CSF) routine examination, CSF chemistry tests, and immunological tests, have low specificity or sensitivity. While, microbiological culture is considered the reference standard, it is time-consuming and has low sensitivity [6]. CSF molecular tests, on the other hand, have high specificity but consistently low sensitivity [7]. As a result, the ability to differentiate CNS infections, common bacterial meningitis (BM), tuberculous meningitis (TBM), and cryptococcal meningitis (CM), is currently limited. Therefore, there is an urgent need to explore biomarkers that can aid in the differential diagnosis of CNS infections [8].

Neutrophil Gelatinase-Associated Lipocalin (NGAL), also known as human neutrophil lipocalin (HNL) or lipocalin 2, was initially discovered and purified from neutrophils [9]. NGAL is stored in the secondary granules of neutrophil granulocytes but it is also produced by other cells and tissues [10]. It exists in different forms, including as a monomer, homodimer, and heterodimer [11]. As an emerging biomarker of acute kidney injury, serum NGAL has been applied to clinical practice; however, its exact biological function remains unclear [12]. Recently, associations of NGAL with diseases have been actively studied in various research fields, including inflammatory disorders [13], cancer [14], cardiovascular disease [15], and neuropsychiatry [16]. Many studies have suggested that NGAL can serve as an early inflammatory biomarker to differentiate between acute bacterial or viral infections [17, 18]. Serum NGAL levels have shown to increase in the presence of a bacterial infection, with higher sensitivities and specificities compared to conventional inflammatory markers [19]. Additionally, CSF NGAL concentrations have been measured in some studies, which are closely associated with the pathogenesis of diseases [20, 21]. However, it is still unclear whether the CSF NGAL is changed during the CNS infection and it can be a potential and reliable biomarker for differentiating between BM and TBM or CM.

In the present study, we prospectively enrolled CNS infection patients, including BM, TBM, CM, and hospitalized controls (HC) with no CNS infection and normal CSF examination findings, to detect CSF NGAL levels and evaluate the value of CSF NGAL in distinguishing BM from two other clinically common types of meningitis (TBM and CM), as well as its significance in monitoring antibiotic therapy for BM.

## Materials and methods

### Study design and participants

In the current study, we prospectively enrolled patients with suspected CNS infections, in the First Affiliated Hospital of Fujian Medical University (Fuzhou, China) from May 2019 to June 2022. The study protocol was approved by the Institutional Review Board of the First Affiliated Hospital of Fujian Medical University (No, MRCTA, ECFAH of FMU, [2020]265). Written informed consent was obtained from participants or their surrogates prior to the study.

The case inclusion criteria were as follows: (1) clinical features were characterized by headache, fever, vomiting, neck stiffness, or meningeal irritation signs; (2) The presence of abnormalities in routine CSF examinations and CSF chemistry tests (such as elevated white blood cells and proteins) suggests the presence of intracranial infection. (3) Patients who cooperated with the study and were willing to sign informed consent. Ultimately, these patients were categorized into four groups: BM, TBM, CM, and others, based on the final diagnosis made by at least two experienced neurologists at the time of discharge. The diagnosis of BM, TBM, and CM was based on the following criteria: (1) Confirmed BM was diagnosed based on the positive results for at least one of the following tests: culture of pathogenic bacteria, PCR, or mNGS analysis from CSF. Probable BM was diagnosed based on clinical symptoms, typically abnormal CSF findings, and a good response to antibiotics. The criteria for the convalescent period of BM were as follows: After antibiotic treatment for more than 7 days, the clinical symptoms of BM patients significantly improved, and the levels of the CSF white blood cells and proteins decreased significantly or have returned normal levels compared to the initial period of the onset of the disease. (2) The definite TBM was diagnosed by the culture-positive presence of M.tb or the positive acid-fast staining in the CSF. The probable TBM was diagnosed based on clinical manifestations and CSF findings, including the following changes: a history of or exposure to tuberculosis; positive interferon gamma release assays in the blood; effective anti-TB treatment; and the exclusion of alternative diagnoses [22]. (3) The confirmed CM was diagnosed by the positive CSF india ink or the Cryptococcus neoformans seen by May-Grunwald-Giemsa (MGG) staining in the CSF. Additionally, the controls were from those patients who excluded the CNS infection with normal CSF examination findings. The others who were suffering from chronic liver disease, especially kidney disease, other tumors, and a shorter survival time were excluded.

### Collection of sample and patient’s data

Prospectively collected baseline data on demographics, clinical signs and symptoms at the time of admission, and general examination, including blood routine tests, CSF common examinations, and serum white blood counts (WBC) and C reactive protein (CRP).

All enrolled patients underwent lumbar punctures at the L4 and L5 levels by the physicians after admission, and 3 mL of CSF were extracted into a sterile tube. After centrifugation at 2000 g for 5 min, CSF supernatant samples were collected and stored at −80 °C until analysis. The paired serum specimens from each subject were collected simultaneously, centrifuged at 1,500 g for 10 min, and stored at −80 °C until used. Furthermore, in order to observe the dynamic change of CSF NGAL, the CSF and paired serum specimens collected during the convalescent period of some BM patients (from March 2021 to June 2022) were also collected.

### The measurement of NGAL in CSF and serum

The CSF and serum NGAL were measured by an automatic biochemistry analyzer (Siemens, ADVIA2400), using a commercialized kit (the Latex enhanced immunoturbidimetry methods), which have been approved by the CFDA, from Wuhan Life Origin Biotech Joint Stock Co., Ltd.

### Statistical analysis

Data analysis was performed with SPSS 18.0 software (Chicago, USA). Continuous variables that did not have a normal distribution were expressed as medians and interquartile ranges (IQR). The non-parametric test (Kruskal–Wallis test or Mann–Whitney test) was used to compare outcomes. Categorical variables were expressed as the number (%) and compared using the Chi-square test. For paired comparisons, Wilcoxon’s non-parametric test for paired samples was used. The diagnostic accuracy of the biochemical markers was assessed by receiver operating characteristic (ROC) curve analysis. The Spearman correlation coefficient was employed to evaluate the correlations. All tests of significance were two-tailed, and *P* < 0.05 was considered statistically significant.

## Results

### Characteristics of studying subjects

**Fig. 1:**
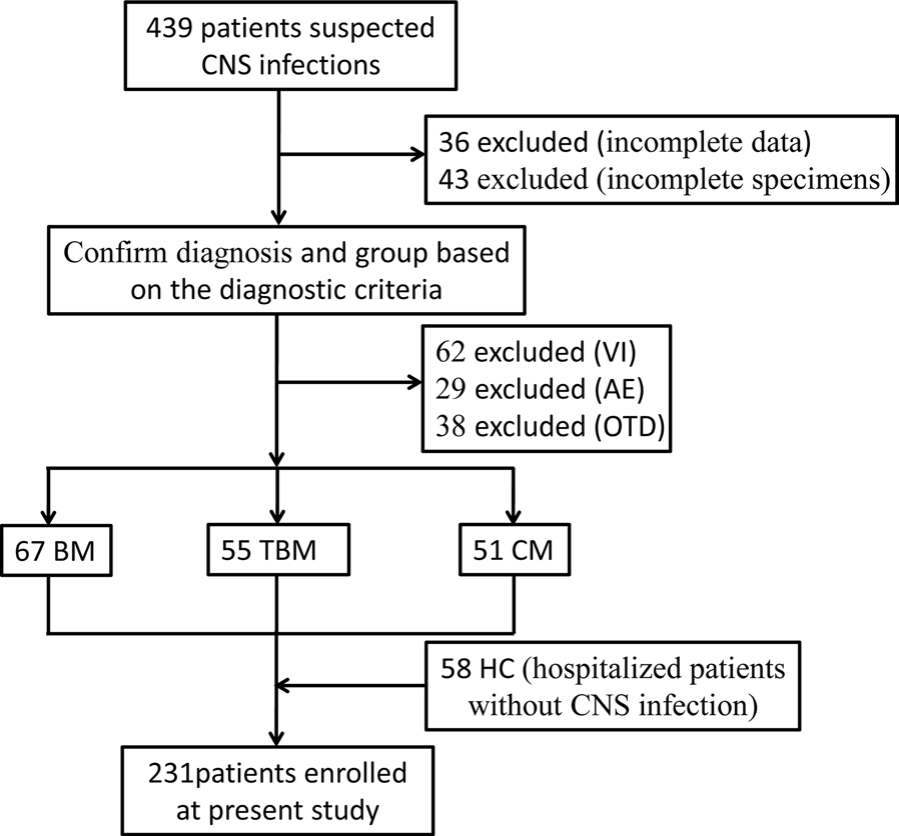
Flowchart of the inclusion and exclusion of participants in this study. *CNS* central nervous system, *VI* viral infection, *AE* autoimmune encephalitis, *OTD* final diagnosis as other diseases, *BM* bacterial meningitis, *TBM* tuberculous meningitis, *CM* cryptococcal meningitis, *HC* hospitalized controls

In the current study, we prospectively obtained 439 patients with suspected CNS infections at admission (Fig. 1). After the later evidence, including the data of diagnosis and therapy, we preliminary excluded 36 patients whose clinical data were not completely obtained and 43 patients whose specimens at the submission were not completed. Meanwhile, viral infections (VI), autoimmune encephalitis (AE), and other patients who were confirmed to have other diseases (OTD) were excluded. Based on the diagnostic criteria in Methods, we further divided residual patients into three case groups: the BM group (n = 67), the TBM group (n = 55) and the CM group (n = 51). Additionally, 58 patients who were hospitalized and excluded from CNS infections with normal CSF examination findings were used as controls (HC).

The demographic and clinical characteristics of subjects at admission are summarized in Table 1. There were no significant differences among the four groups in terms of demographic characteristics (*P* > 0.05). The most common symptoms presented in three case groups were fever and headache, and some patients also presented with vomiting and altered consciousness. The ratio of fever in the TBM was 85.4%, higher than the BM (73.1%) and CM (66.7%), and the ratio of headache in the CM was 100%, which was higher than the TBM (92.7%) and BM (83.6%); however, there were no significant differences (*P* > 0.05). On physical examination, the positive rate of meningeal irritation (including neck stiffness, Kernig sign, and Brudzinski sign) was highest in the TBM (70.9%), followed by the BM (61.2%) and the CM (56.8%), which also had no significant differences (*P* > 0.05). The intracranial pressure (IP) in the CM was highest, followed by the BM and the TBM, which were significantly higher than the HC.

**Table 1:** Clinical characteristics and common laboratory tests of the study subjects

Characteristics	BM (n = 67)	TBM (n = 55)	CM (n = 51)	HC (n = 58)	*P* value
Age, median(range)	43 (0.8–84)	48 (27–80)	41 (14–73)	48 (22–81)	0.093
Male, n (%)	44 (65.7)	38 (69.1)	37 (72.5)	34 (58.6)	0.336
Fever, n (%)	49 (73.1)	47 (85.4)	34 (66.7)	–	0.271
Headache, n (%)	56 (83.6)	51 (92.7)	51 (100)	–	0.355
Vomiting, n (%)	20 (29.8)	13 (23.6)	12 (23.5)	–	0.833
Altered consciousness, n (%)	11 (16.4)	7 (12.7)	8 (15.6)	–	0.856
Meningeal irritation, n (%)	41 (61.2)	39 (70.9)	29 (56.8)	–	0.279
IP (mmH2O), median (IQR)	230 (103,350)	190 (60,330)	270 (165,360)	157 (50,220)	0.402
CSF smear/culture, n (%)	9 (13.4)	1 (1.8)	37 (72.5)^†^	–	< 0.001
ATBA n (%)	58 (86.6)	40 (72.7)	36 (70.6)	21 (63.6)	0.784
CSF, median (IQR)					
WBC(× 10^6^/L)	228 (90,1977)	173 (65,425)	107 (54,176)	3.7 (3.0,6.0)	< 0.001
Chloride(m mol/L)	114 (112,118)	113 (108,120)	115 (109,121)	121 (119,123)	0.011
Glucose(m mol/L)	2.58 (2.01,3.02)	2.43 (1.93,2.62)	2.46 (1.91,2.66)	2.98 (2.65,3.49)	0.001
Protein(g/L)	1.42 (0.88,2.93)	1.38 (0.72,2.13)	1.23 (0.73,1.80)	0.41 (0.30,0.51)	< 0.001
Serum, median (IQR)					
WBC (× 10^9^/L)	11.4 (4.5,21.2)	8.7 (5.1,14.7)	10.7 (8.6,16.8)	7.6 (3.8,18.7)	0.039
CRP (g/L)	108.2 (2.6,401)	74.5 (1.9,253)	113.6 (5.0,330)	56.6 (2.2,160)	0.351

Data were expressed as median and interquartile range (IQR) or the number (%). The Kruskal–Wallis test (Mann–Whitney U test) or Chi-squared test were applied to compare the outcomes as appropriate

*BM* bacterial meningitis, *TBM* tuberculous meningitis, *CM* cryptococcal meningitis, *HC* hospitalized controls, *IP* Intracranial pressure, *ATBA* Antibiotic therapy before admission, *WBC* White blood count

^†^The Cryptococcus can be seen by May- Grunwald-Giemsa (MGG) staining or india ink in the CSF

### Laboratory characteristics of studying subjects

To evaluate the discriminatory ability of common CSF and serum parameters for different etiologies, we conducted a preliminarily comparison of the findings from common CSF laboratory tests (WBC, chloride, glucose, and protein) and serum WBC and CRP in patients in four groups at admission. As shown in Table 1, the CSF WBC in three case groups was significantly higher than the HC group (all *P* < 0.01), however, there were no significant differences among the three case groups (all *P* > 0.05; Additional file 1: Figure S1A). Meanwhile, the CSF Chloride and Glucose in BM, TBM, and CM were lower than the HC (all *P* < 0.05), but there were also no significant differences among the three case groups (all *P* > 0.05; Additional file 1: Figure S1B and C). Although the CSF protein was higher in BM (median and IQR: 1.42 (0.88–2.93) mg/L) than TBM (median and IQR: 1.38 (0.72–2.13) mg/L) or CM (median and IQR: 1.23 (0.73–1.80) mg/L), there were no significant differences (*P* > 0.05; Additional file 1: Figure S1D). Moreover, the serum WBC in the case groups were significantly different from the HC group (*P* < 0.05), however, there was no significant difference among the three case groups. In addition, the serum CRP in the three case groups was not significantly different from the HC group (Table 1).

### The levels of CSF and serum NGAL of studying subjects

**Fig. 2:**
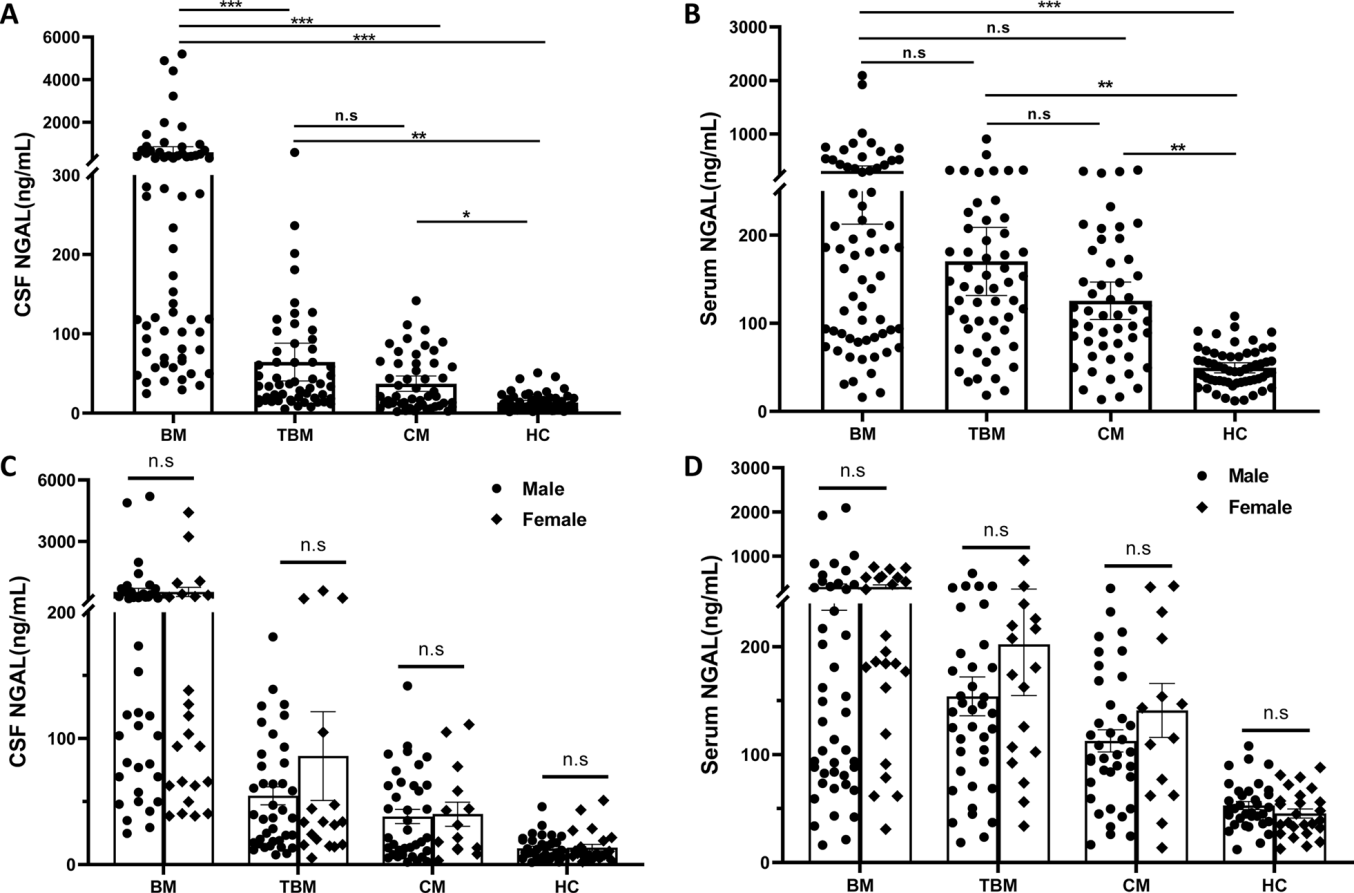
The levels of NGAL in CSF and serum among the four groups. **A**, the levels of CSF NGAL among the patients with BM, TBM, CM, and HC; **B**, the serum NGAL among the patients with BM, TBM, CM, and HC. **C**, the levels of CSF NGAL between males and females among the patients with BM, TBM, CM, and HC; **D**, the levels of serum NGAL between males and females among the patients with BM, TBM, CM, and HC. *NGAL* Neutrophil Gelatinase-Associated Lipocalin, *BM* bacterial meningitis, *TBM* tuberculous meningitis, *CM* cryptococcal meningitis, *HC* hospitalized controls. *P < 0.05; **P < 0.01, ***P < 0.001, compared with the BM group. *n.s* not significant

Next, we examined the levels of CSF and serum NGAL among the BM, TBM, CM and HC in the acute period (at admission). The CSF NGAL in BM (median and IQR: 253.36 (79.81–535.49) ng/mL) was significantly higher than TBM (median and IQR: 35.89 (17.27–80.61) ng/mL), CM (median and IQR: 21.25 (10.91–62.58) ng/mL), and HC (median and IQR: 9.28 (6.11–18.84) ng/mL), as shown in Fig. 2A (all *P* < 0.05). Later, we also detected the serum NGAL. We found that the serum NGAL in three case groups: BM (median and IQR: 180.93 (83.90–378.81) ng/mL), TBM (median and IQR: 141.61 (92.29–207.61) ng/mL), and CM (median and IQR: 109.38 (74.27–172.38) ng/mL) were higher than HC (median and IQR: 46.01 (34.75–63.01) ng/mL); however, there were no significant differences among BM, TBM, and CM (Fig. 2B, all *P* > 0.05).

To evaluate the effects of gender on NGAL levels, we compared CSF and serum NGAL levels in males and females in the BM, TBM, CM, and HC groups, respectively. As shown in Fig. 2C, the CSF NGAL showed no significant differences between males and females both in BM, TBM, CM, and HC (all *P* > 0.05). Similarly, no significant differences were found in serum NGAL between males and females in BM, TBM, CM, and HC (Fig. 2D, all *P* > 0.05).

### Diagnostic accuracy of CSF NGAL for acute BM

**Fig. 3:**
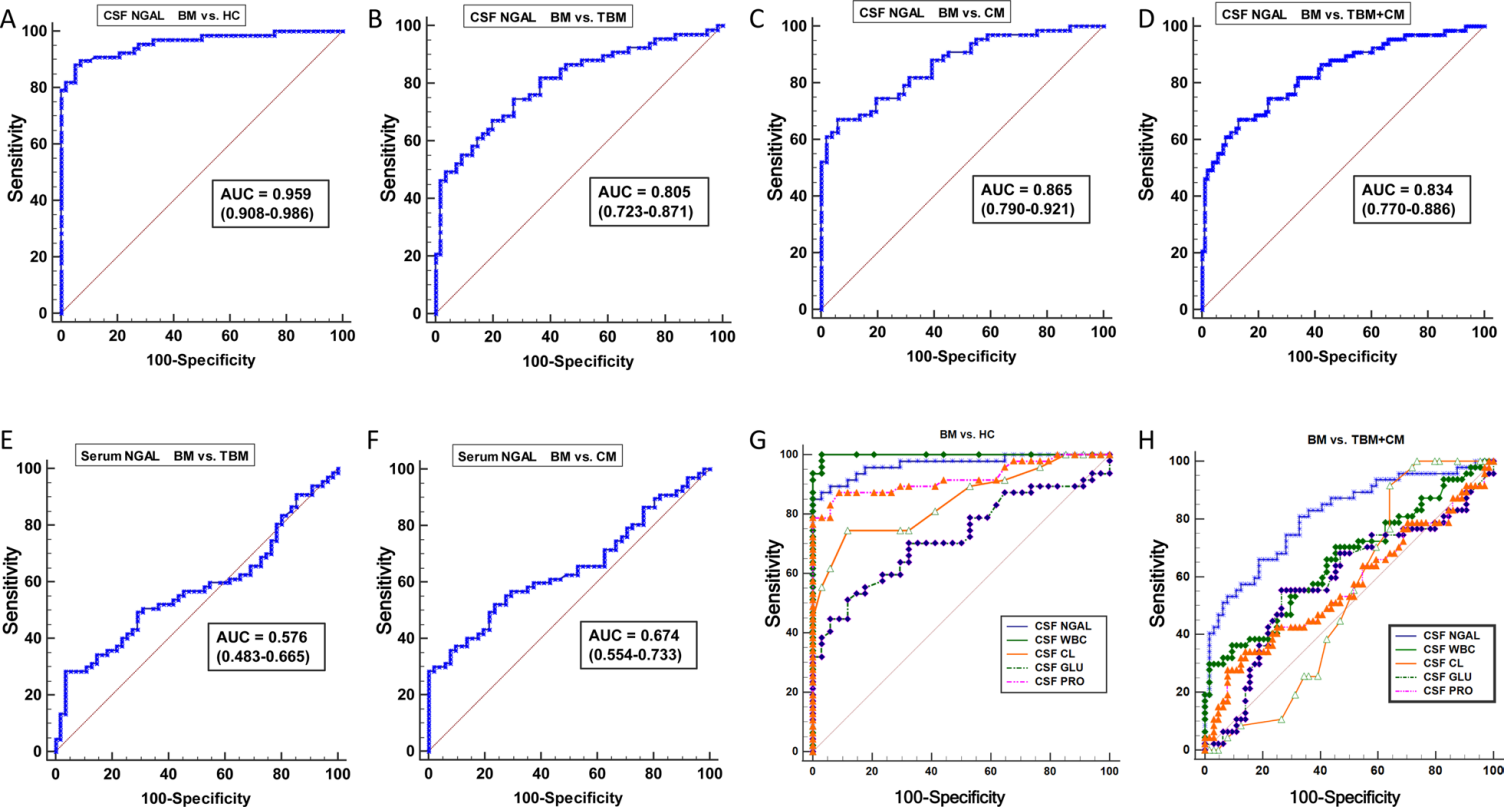
ROC curve analysis of CSF NGAL and serum NGAL for the diagnosis of acute bacterial meningitis. **A**, ROC curve of CSF NGAL for discriminating patients with BM (n = 67) from HC (n = 58) with an AUC of 0.955 (0.903–0.984; *P* < 0.001). **B**, ROC curve of CSF NGAL for discriminating patients with BM (n = 67) from TBM (n = 55) with an AUC of 0.805 (0.723–0.871; *P* < 0.01). **C**, ROC curve of CSF NGAL for discriminating patients with BM (n = 67) from CM (n = 51) with an AUC of 0.865 (0.790–0.921; *P* < 0.01). **D**, ROC curve of CSF NGAL for discriminating patients with BM (n = 67) from TBM (n = 55) and CM (n = 51) with an AUC of 0.834 (0.770–0.886; *P* < 0.01). **E**, ROC curve of serum NGAL for discriminating patients with BM (n = 67) from TBM (n = 55) with an AUC of 0.576 (0.483–0.665; *P* > 0.05). **F**, ROC curve of serum NGAL for discriminating patients with BM (n = 67) from CM (n = 51) with an AUC of 0.674 (0.554–0.733; *P* = 0.035). **G**, The ability of CSF NGAL and traditional CSF assays (CSF WBC, chloride, glucose, and protein) to differentiate between patients with BM (n = 67) and HC (n = 58) was evaluated using ROC curve analysis. **H**, The ability of CSF NGAL and traditional CSF assays (CSF WBC, chloride, glucose, and protein) to discriminate patients with BM (n = 67) from TBM (n = 55) and CM (n = 51) was evaluated using ROC curve analysis. *NGAL* Neutrophil Gelatinase-Associated Lipocalin, *BM* bacterial meningitis, *TBM* tuberculous meningitis, *CM* cryptococcal meningitis, *HC* hospitalized controls, *WBC* White blood count, *CL* chloride, *GLU* glucose, *PRO* protein. *P < 0.05; **P < 0.01, ***P < 0.001, compared with the BM group

In order to further evaluate the diagnostic value of CSF NGAL or serum NGAL for BM, we used the ROC analysis to calculate the area under the curve (AUC), cutoff value, sensitivity, and specificity, respectively. Our results showed that the CSF NGAL exhibited a larger AUC (0.955), with a 95% confidence interval [CI] of 0.903 to 0.984 (*P* < 0.001) than the serum NGAL, in which the AUC was 0.724 and the 95% CI was 0.587 to 0.861. At a CSF NGAL cutoff value of 31.03 ng/mL, there was the optimal sensitivity (88.06%) and specificity (93.01%) for distinguishing the BM and HC (Fig. 3A). Meanwhile, the AUC of CSF NGAL for discriminating BM with TBM is 0.812 (0.723–0.876) with 74.63% sensitivity and 74.14% specificity (Fig. 3B and Table 2); and the AUC for discriminating BM with CM is 0.865 (0.790–0.921) with 74.36% sensitivity and 80.39% specificity (Fig. 3C and Table 2). More importantly, the AUC of CSF NGAL for discriminating BM from TBM and CM is 0.834 (0.770–0.886) with 70.15% sensitivity and 77.36% specificity (Fig. 3D and Table 2). Later, we evaluated the diagnostic value of serum NGAL for discriminating BM with TBM or CM. While serum NGAL exhibited poor diagnostic ability for discriminating BM with TBM (Fig. 3E) or CM (Fig. 3F).

**Table 2:** The diagnostic efficacy evaluation of CSF NGAL for acute BM

CSF NGA L	AUC-ROC (95%CI)	Cutoff (ng/mL)	Sensitivity (95% CI)	Specificity (95% CI)	*P* value
BM vs.TBM	0.812 (0.723–0.876)	64.24	74.14 (62.5–84.5)	74.14 (61.0–84.7)	< 0.001
BM vs.CM	0.724 (0.587–0.861)	65. 23	74.36 (64.5–84.5)	80.39 (66.9–90.2)	< 0.001
BM vs.TBM + CM	0.834 (0.770–0.886)	74.27	70.15 (57.7–80.7)	77.36 (68.2–84.9)	< 0.001

*BM* bacterial meningitis, *AUC* area under the curve, *ROC* receiver operating characteristic curve, *NGAL* Neutrophil Gelatinase-Associated Lipocalin, *CI* confidence interval

To further analyze the superiority of CSF NGAL in diagnosing BM compared to traditional CSF assays, we conducted ROC curve analysis to compare the accuracy of CSF NGAL with CSF WBC, CSF chloride, glucose, and protein for distinguishing BM and HC. Our findings showed that CSF NGAL was slightly less accurate than CSF WBC in differentiating BM from healthy controls (Fig. 3G; *P* < 0.05); however, it was significantly better than CSF chloride, glucose, and protein (Fig. 3G; all *P* < 0.05). We also focused on comparing the diagnostic efficacy of CSF NGAL in discriminating BM from TBM and CM. Our results demonstrated that the diagnostic efficacy of CSF NGAL in this differential diagnosis was significantly better than that of the traditional CSF assays (CSF WBC, chloride, glucose, and protein), and CSF NGAL exhibited superior AUC, sensitivity, and specificity compared to the above four assays (Fig. 3H; all *P* < 0.05).

### Associations between CSF and serum NGAL and other traditional markers

**Fig. 4:**
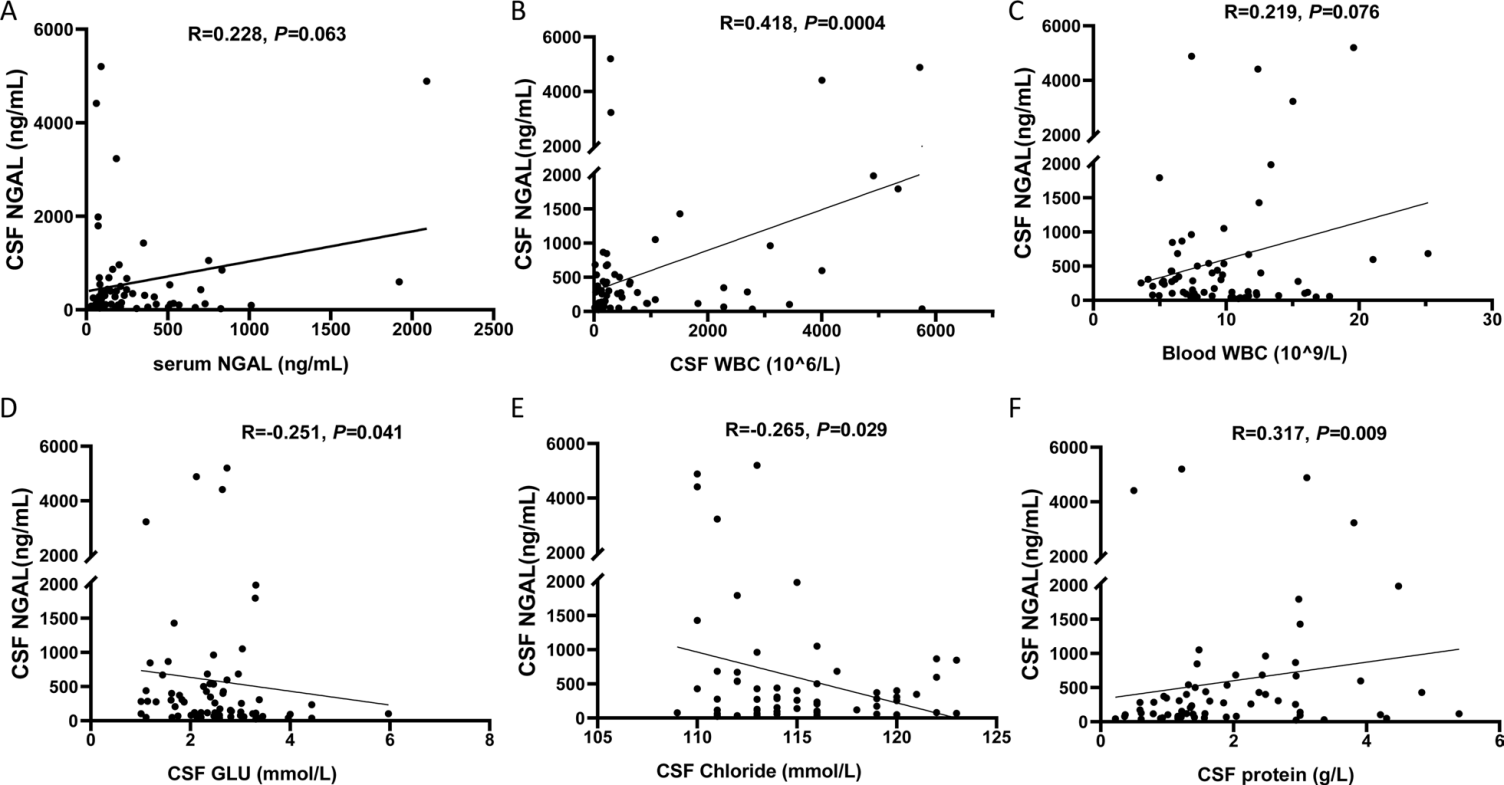
Correlation analysis of CSF and serum NGAL and other traditional markers. Spearman correlation analysis was carried out to evaluate the correlation of CSF NGAL in BM with other clinical parameters. **A**, Correlation between the levels of CSF NGAL and serum NGAL in BM patients (n = 67). **B**, Correlation between the levels of CSF NGAL and CSF WBC in BM patients (n = 67). **C**, Correlation between the levels of CSF NGAL and blood WBC in BM patients (n = 67). **D**, Correlation between the levels of CSF NGAL and CSF glucose in BM patients (n = 67). **E**, Correlation between the levels of CSF NGAL and CSF chloride in BM patients (n = 67). **F**, Correlation between the levels of CSF NGAL and CSF protein in BM patients (n = 67). *NGAL* Neutrophil Gelatinase-Associated Lipocalin, *BM* bacterial meningitis, *TBM* tuberculous meningitis, *CM* cryptococcal meningitis, *HC* hospitalized controls

First, we evaluated the associations between CSF NGAL and serum NGAL. Our results showed there was no significant correlation between the levels of CSF NGAL and serum NGAL (*P* > 0.05, Fig. 4A). Then, we analyzed the associations between CSF NGAL and CSF and blood WBC, respectively. Our results showed the CSF NGAL was positively correlated with the CSF WBC (R = 0.418, *P* = 0.0004; Fig. 4B), while no significant correlation was found between the CSF NGAL and blood WBC (R = 0.219, *P* = 0.075; Fig. 4C). What’s more, we also evaluated the associations between CSF NGAL and conventional CSF tests, including CSF glucose, chloride, and protein. Our results showed that the CSF NGAL was negatively correlated with the CSF glucose (R = −0.251, *P* = 0.041; Fig. 4D) and CSF chloride (R = −0.265, *P* = 0.029; Fig. 4E) and positively correlated with CSF protein (R = 0.317, *P* = 0.008; Fig. 4F).

### The monitoring value of CSF NGAL for the antibacterial treatment

**Fig. 5:**
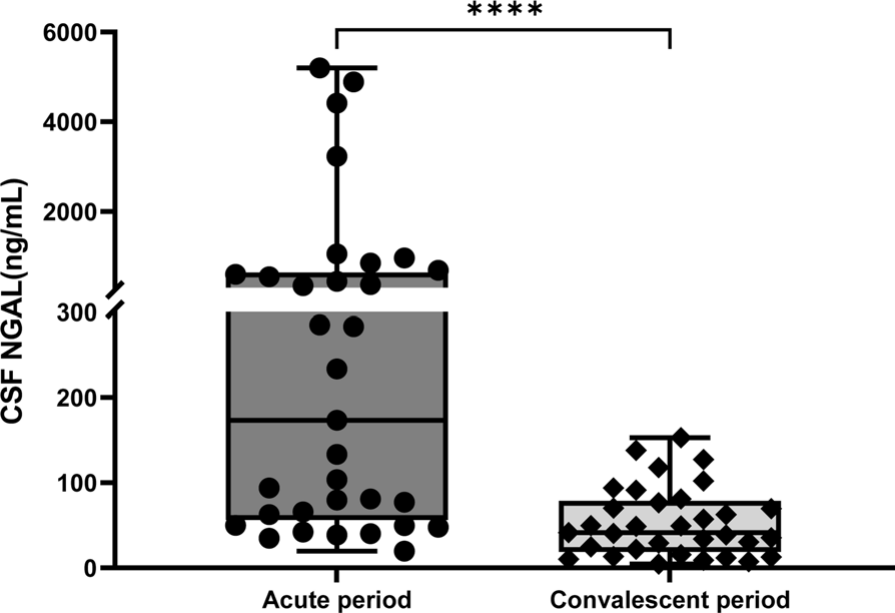
CSF levels of NGAL in patients with acute and convalescent bacterial meningitis. *NGAL* Neutrophil Gelatinase-Associated Lipocalin. ****P < 0.0001, compared with the acute period

To evaluate whether the CSF NGAL would be a useful monitor of the antibacterial treatment, we also collected CSF samples of BM both in the acute and convalescent periods, and longitudinally measured the CSF NGAL. As shown in Fig. 5, the CSF NGAL in the convalescent period (median and IQR: 41.50 (18.78–52.98) ng/mL) was significantly decreased compared to the acute period (median and IQR: 173.36 (51.68, 640.46) ng/mL).

## Discussion

In the current study, we found the CSF NGAL in patients with BM was significantly higher than those with TBM, CM, and HC, and the CSF NGAL would be serve as a novel and superior biomarker to distinguish BM from those with suspected CNS infection, particularly when a differential diagnosis is needed from patients with TBM or CM. Additionally, our findings indicated that CSF NGAL was a potential monitoring marker for the antibacterial treatment.

The suspicion of CNS infection is a daily concern in clinical practice [3]. Because the clinical manifestations of BM, TBM, and CM are similar and the common diagnostic technologies are insensitive or non-specific, it is still challenging and difficult to make an early differential diagnosis. Recently, lots of studies have been performed to search for reliable biomarkers to differentially determine the etiology of CNS infection, such as CSF PCT [23], CSF lactate [24], CSF cytokines [25], and CSF heparin binding protein [26]. Although some biomarkers have shown great potential for discriminating between bacterial or viral infections, few have been valuable for the differential diagnosis of BM with TBM or CM.

In the present study, we prospectively enrolled patients suspected of having meningitis and divided them into three case groups (BM, TBM, and CM) and a hospitalized control group (HC). Firstly, we preliminary compared the common CSF laboratory parameters among the patients with BM, TBM, and CM to evaluate their differential diagnosis value. Our results showed that compared with HC, increased counts of WBC, low glucose, and high protein concentrations of CSF were observed in three case groups; however, there were no significant differences among BM, TBM, and CM. These results indicated the common CSF laboratory tests can preliminary differentiate the CNS infection from non-infection but cannot differentiate the BM from TBM or CM, which was consistent with the previous studies [27].

NGAL was initially identified in neutrophils and is primarily produced by damaged nephron epithelia, although it is also generated by macrophages, active leukocytes, the choroid plexus, and a variety of other tissues [28, 29]. Apart from being a biomarker of renal injury, more and more evidence has been reported that NGAL is an acute-phase protein and plays a role in innate immunity, particularly in the early stages of infection. Recent studies have also reported its neuroprotective effects during neuroinflammatory processes [30]. In the current study, we wondered whether the levels of NGAL in the CSF were different in patients with BM, TBM, or CM and whether the CSF NGAL could be used as a biomarker for distinguishing BM from TBM or CM. Our results showed the CSF NGAL in BM was significantly higher than in TBM and CM. Although the serum NGAL in BM was higher than HC, there were no significant differences among BM, TBM, or CM. Importantly, CSF NGAL demonstrated a strong diagnostic performance in differentiating acute BM from TBM and CM, surpassing the diagnostic efficacy of traditional CSF tests. Conversely, serum NGAL exhibited limited diagnostic ability. These results suggested the CSF NGAL has the potential to be a biomarker for further discriminating BM from TBM or CM.

Serum NGAL has been utilized as a biomarker for clinical acute kidney injury patients. Per Venge et al. have proposed that serum NGAL can also be employed for the timely and early diagnosis of bacterial infections, similar to CRP [13]. Common immunological methods can automatically detect serum and CSF NGAL. Furthermore, the cost of NGAL detection is economical in comparison to molecular biology techniques like next-generation sequencing. Consequently, CSF NGAL holds potential for timely and cost-effective identification of BM patients, similar to traditional CSF chemistry tests.

Moreover, previous studies have reported gender-dependent signatures for NGAL, with its expression being linked to metabolic and cardiovascular problems in males [31, 32]. In a recent study by Jover et al., it was found that NGAL expression is higher in males than females in patients with aortic stenosis. This elevated expression was associated with inflammation, oxidative stress, osteogenesis, and calcific load [33]. In light of these findings, we also investigated the impact of gender on NGAL expression in both CSF and serum. Our results indicate that there were no significant differences in CSF NGAL or serum NGAL levels between males and females in the four groups (BM, TBM, CM, and HC). To the best of our knowledge, no gender differences in CSF NGAL have been reported thus far. Our findings suggest that the levels of CSF NGAL do not exhibit significant gender differences in the context of intracranial infections. Therefore, future studies should consider different pathological conditions of the disease and tissue specificity when exploring sex differences in NGAL expression. Of course, our results also need further validation in future research.

The exact regulatory mechanisms of NGAL are still insufficiently understood. As a component of the innate immune response during acute neuroinflammation, NGAL is produced at the blood brain barrier by the choroid plexus epithelial cells and the endothelial cells of blood vessels [34]. To explore the potential mechanisms of CSF NGAL during bacterial meningitis, we evaluated the association between CSF NGAL and serum NGAL and other CSF conventional tests, including CSF WBC, CSF glucose, chloride, and protein. According to our present results, the CSF NGAL in BM was higher and more persistent than the serum NGAL. Although there was a significant correlation between the levels of CSF NGAL and CSF conventional parameters, the correlation coefficients were relatively lower. These data indicated that the elevated CSF NGAL may not originate from the peripheral blood and may not be directly produced by neutrophils in the CSF. We speculated that the elevated CSF NGAL in patients with BM may be due to increased synthesis in the choroidal epithelia and endothelial cells of blood vessels.

Apart from the differential diagnosis of BM in the acute period, the monitoring of antibiotic treatment is also an important clinical challenge. To further evaluate whether the CSF NGAL would be a useful monitor of the antibacterial treatment, we longitudinally measured the CSF NGAL of BM in the acute and convalescent periods. We found the CSF NGAL in the convalescent period of BM was significantly decreased compared to the acute period. Our study’s findings are consistent with those of other studies of serum NGAL [17]. Thus, CSF NGAL would also be used as a sign of therapeutic success and a potential biomarker for monitoring antibiotic therapy in BM.

Although previous studies have reported the significance of CSF NGAL in BM [35, 36], the current study used a prospective cohort study to clarify the value and feasibility of CSF NGAL for the early differential diagnosis of BM from two other clinically common types of meningitis (TBM and CM), based on the actual challenges in clinical. The study not only focused on the expression of NGAL in acute-phase BM, but also investigated the changes of CSF NGAL in patients with convalescent BM, and explores its potential in monitoring antibiotic therapy for BM patients. Furthermore, this study delves into the mechanisms underlying the production and origin of CSF NGAL in BM by analyzing the correlation between CSF NGAL and serum NGAL, as well as examining the differences in its expression between males and females, thereby providing valuable insights for future research.

However, there were some limitations that should be pointed out. First, due to the limitations of diagnostic technologies, not all of the patients in the current study had an etiological diagnosis, which would cause an incorporation bias. Second, because most of the patients in our study were transferred from a primary health care setting where they had accepted empirical antibiotic therapy before admission, we could not get data on the CSF NGAL before antibiotic therapy. Third, according to previous studies, the monomeric form and, to some extent, the heterodimeric forms of NGAL are mainly produced by tubular epithelial cells, while the homodimeric forms are specific to neutrophils [37, 38]. However, the present methodologies for NGAL could not identify the precise forms of NGAL, which may help determine the origin of CSF NGAL.

## Conclusions

In conclusion, our study has suggested that CSF NGAL might be used as an early diagnostic biomarker of BM, especially for differential diagnosis with patients with TBM or CM during the acute periods, and it also might have some important clinical value for monitoring antibiotic therapy for BM. Further research is required to identify the origin of increased CSF NGAL and to determine whether it has a bacteriostatic properties or is merely a marker during the pathogenesis of BM. This will contribute to a more precise understanding of NGAL's role in the pathogenesis of BM and its potential clinical applications.

### Supplementary Information

**Additional file 1: Figure S1.** The levels of traditional CSF assays (CSF WBC, chloride, glucose, and protein) among the four groups. A, the levels of CSF WBC among the patients with BM, TBM, CM, and HC; B, the levels of CSF chloride among the patients with BM, TBM, CM, and HC; C, the levels of CSF glucose among the patients with BM, TBM, CM, and HC; D, the levels of CSF protein among the patients with BM, TBM, CM, and HC. WBC, White blood count; NGAL, Neutrophil Gelatinase-Associated Lipocalin; BM, bacterial meningitis; TBM, tuberculous meningitis; CM, cryptococcal meningitis; HC, hospitalized controls. ** P < 0.01, *** P < 0.001, compared with the HC group. n.s, not significant.

## Data Availability

The data that support the findings of this study are available from the corresponding author upon reasonable request.
