# Supplementary figures and images for: Diagnostic value of cerebrospinal fluid Neutrophil Gelatinase-Associated Lipocalin for differentiation of bacterial meningitis from tuberculous meningitis or cryptococcal meningitis: a prospective cohort study
Source: J Transl Med. 2023 Sep 7;21:603. doi: 10.1186/s12967-023-04485-w (PMC10486126; doi:10.1186/s12967-023-04485-w)

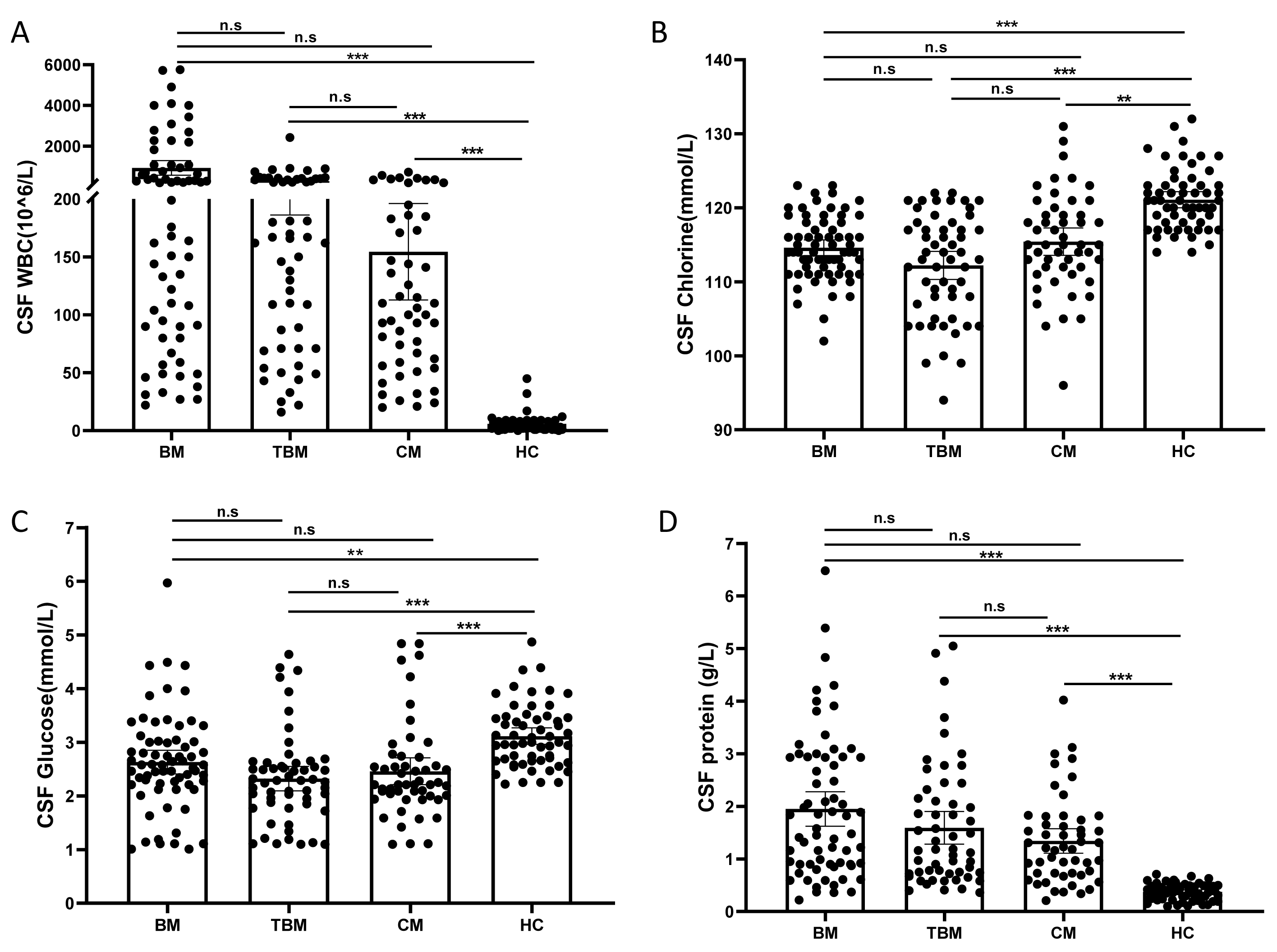

Supplement: Supplementary file 1 — Additional file 1: Figure S1. The levels of traditional CSF assays (CSF WBC, chloride, glucose, and protein) among the four groups. A, the levels of CSF WBC among the patients with BM, TBM, CM, and HC; B, the levels of CSF chloride among the patients with BM, TBM, CM, and HC; C, the levels of CSF glucose among the patients with BM, TBM, CM, and HC; D, the levels of CSF protein among the patients with BM, TBM, CM, and HC. WBC, White blood count; NGAL, Neutrophil Gelatinase-Associated Lipocalin; BM, bacterial meningitis; TBM, tuberculous meningitis; CM, cryptococcal meningitis; HC, hospitalized controls. ** P < 0.01, *** P < 0.001, compared with the HC group. n.s, not significant. [file 12967_2023_4485_MOESM1_ESM.tif]
